# The seventh kidney cancer research summit: progress in accelerating cures

**DOI:** 10.1093/oncolo/oyag204

**Published:** 2026-05-24

**Authors:** Toni K Choueiri, A Ari Hakimi, Rana McKay, Sumanta K Pal, Thomas Powles, Kevin Pels, Bryan Lewis, Susan Poteat, Hans Hammers

**Affiliations:** Department of Medical Oncology, Dana-Farber Cancer Institute, Boston, MA 02215, United States; Department of Surgery, Urology Service, Memorial Sloan Kettering Cancer Center, New York, NY 10065, United States; Departments of Medicine and Urology, University of California San Diego, San Diego, CA 92037, United States; Department of Medical Oncology and Therapeutics Research, City of Hope Comprehensive Cancer Center, Duarte, CA 91010, United States; Barts Cancer Institute, Queen Mary University of London, London, EC1M 6BQ, United Kingdom; Department of Medical Oncology, Dana-Farber Cancer Institute, Boston, MA 02215, United States; KidneyCAN, Philadelphia, PA 19118, United States; KidneyCAN, Philadelphia, PA 19118, United States; Division of Hematology-Oncology, University of Texas Southwestern Medical Center, Dallas, TX 75390, United States

**Keywords:** immunotherapy, kidney cancer, renal cell carcinoma

## Abstract

The seventh Kidney Cancer Research Summit was held as a hybrid virtual/in-person event in Boston, MA during July 17-18, 2025. This conference, which aims to help guide the design of kidney cancer clinical trials and research, centers around projects that showcase the most impactful basic, translational, and clinical data evolving in renal cell carcinoma (RCC), and brings together clinical oncologists, physician scientists, translational researchers, and patient advocates. The abstract presentations were published as a supplement in *The Oncologist* (https://academic.oup.com/oncolo/issue/30/Supplement_2). This perspective will provide the layout of the current RCC research landscape as discussed and debated among the stakeholders at the conference as well as more recent updates.

## Graphical Abstract

**Figure:**
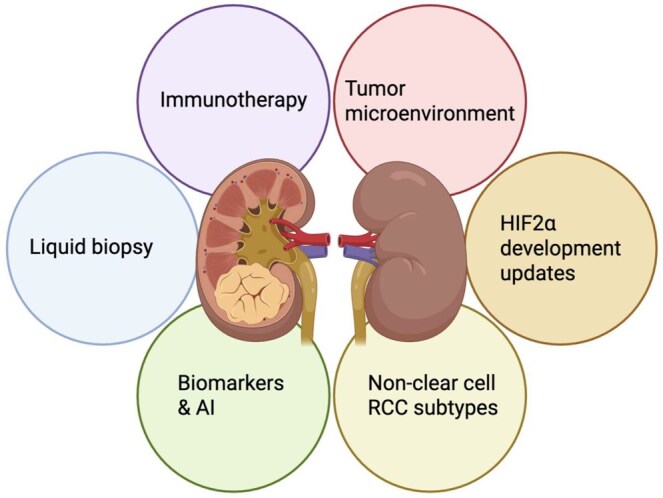

Implications for Practice This article reviews emerging areas of research that are of particular translational and clinical relevance in renal cell carcinoma (RCC). These areas include immunotherapy beyond immune checkpoint inhibitors, features and vulnerabilities of the tumor microenvironment, hypoxia signaling and incorporation of HIF2α inhibitors in clinical practice, and advances in understanding and treating certain rare histological subtypes of RCC. The scope of this article is guided by the discussions and presentations at the Kidney Cancer Research Summit, a yearly conference bringing together key stakeholders in the RCC research community, which provides a view into the most promising current areas of therapeutic development.

## Introduction

In 2026, kidney cancer continues to be one of the 10 most commonly diagnosed malignancies in the United States. According to registries and mortality data, the incidence of renal cancers (80 450) declined slightly from the previous 2 years while the number of deaths (15 160) continues to rise steadily.^1^ Renal cell carcinoma (RCC) is a histologically diverse grouping of cancers of the renal tubular epithelium. Clear cell RCC (ccRCC) is by far the predominant form of the disease, but up to a quarter of cases are variant histologies, and all subtypes may further present with dedifferentiated compartments such as sarcomatoid features. The current treatment paradigm for most subtypes of RCC are combination therapies including at least one immune checkpoint inhibitor (ICI), but many patients do not respond and more still will eventually develop resistance. In 2023, the first-in-class HIF2α inhibitor belzutifan was approved for ccRCC in the post-ICI setting, a bounty of the highly active global clinical and translational research happening in this field. Belzutifan and other novel HIF2α inhibitors are now under investigation in a variety of disease settings, as monotherapy and/or in combinations.^2^ Meanwhile, research into the molecular drivers of variant histology RCC has provided insights into disease biology and identified new targets.

The community of kidney cancer researchers, patients, advocates, and their physicians have organized the Kidney Cancer Research Summit (KCRS) since 2019. The seventh edition focused both on traditional areas of emphasis while also incorporating emerging topics. This perspective will provide the layout of the current RCC research landscape presented and discussed among the stakeholders at the summit, and will update the previous white paper.^3^

## Immunotherapy updates

Most patients with metastatic RCC treated with ICIs are not cured, and many will develop resistance and progress. Two negative phase 3 trials have shown that following progression on ICI-based therapy, there is no evidence-based role for subsequent vascular endothelial growth factor (VEGF) tyrosine kinase inhibitor (TKI)-ICI or dual ICI combinations.^4^^,^^5^ Regulatory T cells (Tregs) expressing PD-1 play an important role in resistance to ICIs and the ratio of PD-1^+^ Tregs to CD8^+^ T cells may be a predictive biomarker for response to anti-PD-1 therapy.^6^ Another study showed that tertiary lymphoid structures and absence of exhausted CD8^+^ T cells are also required for response.^7^ Meanwhile, there is fervent activity looking into prognostic biomarkers for ICI activity/toxicity and identifying other immunotherapy modalities beyond ICIs.

A biomarker of toxicity to ICI therapy was identified from a pan-cancer genomic analysis of immunotherapy trials. A single nucleotide polymorphism in the *IL7* gene is predictive of immune-related adverse events (irAEs) on anti-PD-1 therapy, as well as improved survival.^8^^,^^9^ This biomarker has potential for guiding therapeutic decisions in patients treated with ICIs, including prophylaxis for irAEs.

*SETD2* loss occurs in ∼15% of human ccRCC.^10^ Among other consequences, its loss impairs H3K36 methylation via DNMT3B, increasing expression of cryptic viral repeats otherwise suppressed by SETD2.^11^  *SETD2* loss therefore stimulates immunity and possibly sensitizes tumor cells to ICI,^12^ which could plausibly be mimicked by combining a hypomethylating agent with ICI. However, a small trial of this strategy with guadecitabine + durvalumab showed safety but no added efficacy.^13^

TGFβ1 is a growth factor that drives tumor immune escape across cell types within the tumor microenvironment.^14^ Linavonkibart is an anti-TGFβ1 antibody that traps TGFβ1 in a latent form to prevent signaling, and may help overcome resistance to ICIs. In a phase 1 b study in 30 patients with ccRCC who had previously progressed on anti-PD-1 therapy, linavonkibart + pembrolizumab had a manageable safety profile and multiple responses registered, including one CR.^15^ This combination could be further tested in patients with a high degree of immune infiltration in the tumor microenvironment.

HPK1 is a negative regulator of immune cells, and its inhibition may improve antitumor immunotherapy.^16^ In a phase 1 dose-escalation trial of NDI-101150, a small molecular HPK1 inhibitor, one patient with advanced RCC post-ICI experienced a CR.^17^ Another novel target is the phosphatases PTPN2 & PTPN1, which are central regulators of antitumor immunity in both tumor and immune cells.^18^ A first-in-class oral inhibitor ABBV-CLS-484 is being tested as monotherapy or combination with anti-PD-1 or TKI in a phase 1 b study in solid tumors including ccRCC (NCT04777994).

Decades ago, high-dose IL-2 therapy led to rare but durable complete responses (CRs) despite significant toxicity, and became a disfavored option at the arrival of agents that target the VEGF axis. AB248 is a fusion protein that selectively activates IL-2 pathway in CD8^+^ T cells to avoid pleotropic effects of IL-2 on other cells of the tumor microenvironment.^19^ Another immunostimulatory cytokine being manipulated is IL-18, which tumor cells inactivate by expressing a decoy receptor. ST-067 is a decoy-resistant IL-18 variant that has preclinical activity as monotherapy or in combination with anti-PD-1.^20^^,^^21^

The gut microbiome, specifically states of dysbiosis, can profoundly modulate patient responses to ICIs. Soluble MAdCAM-1 is a plasma protein marker of gut health that associated with improved PFS and OS across 3 clinical cohorts, regardless of therapy.^22^ The live bacterial product CBM588 continues to show promising results, most recently in a small study of patients with metastatic RCC treated with cabozantinib + nivolumab, where the addition of CBM588 improved ORR (14/19 = 74% vs 2/10 = 20%) and 6-month PFS (84% vs 60%).^23^ This builds on previous findings for the combination of nivolumab + ipilimumab + CB588,^24^ which together provide the rationale for continued clinical development of CBM588 with ICI-based regimens.

Personalized cancer vaccines (PCVs) continue to advance as adjuvant therapy. In the phase 1 NEOVAX study peptides derived from tumor antigens specific to each patient with high-risk RCC were generated and delivered, resulting in no disease recurrences during a median follow-up of >3 years and T cell immune response against PCV antigens shown by all 9 patients.^25^ A randomized phase 2 study combining the personalized mRNA vaccine V940 with pembrolizumab has finished accrual (NCT06307431).

## Tumor microenvironment

The adenosine A2 receptor plays a role in metastatic progression and suppression of the T cell-mediated immune response in RCC.^26^ The small molecule A2R inhibitor ciforadenant previously showed activity alone and in combination with ICI against refractory RCC.^27^ In a single-arm first line trial, triplet therapy of ciforadenant with nivolumab/ipilimumab was feasible and well-tolerated, but at interim analysis the triplet was not effective at deepening response rate vs the proven ICI doublet.^28^ One potential path forward for probing adenosine blockade in RCC is to test dual A2A/A2B receptor antagonists that could prove more potent.^29^

BCL-xL is an antiapoptotic protein that is overexpressed in many cancers and associated with metastatic dissemination; in RCC, BCL-XL dependence is marked by mesenchymal cell state.^30^ BCL-xL dependence has since been shown to be a shared feature across RCC subtypes, and the orally bioavailable BCL-xL inhibitor, A-1331852, showed antitumor efficacy in a ccRCC xenograft mouse model.^31^^,^^32^

CD70 is a cell surface marker overexpressed by primary and metastatic ccRCC tumors but not normal renal tissue.^33^ While an antibody-drug conjugate targeting CD70 previously proved ineffective,^34^ 2 different anti-CD70 CAR-T therapies are in early clinical development. CTX130 demonstrated a disease control rate (DCR) above 80% in 16 patients in a dose-expansion phase 1 trial, including one CR^35^; an updated version of this therapy, CTX131, is now being tested.^36^ Another anti-CD70 CAR-T therapy, ALLO-316, has shown activity in heavily pretreated patients with CD70^+^ ccRCC.^37^

ENPP3 is among the most overexpressed receptors on tumor cells in RCC and was previously targeted by the antibody-drug conjugate (ADC) AGS-16C3F, which failed in a phase 2 trial against axitinib in patients with advanced RCC.^38^^,^^39^ A renewed attempt is being made with XmAb819, a bispecific antibody targeting ENPP3 and CD-3 as a strategy to activate T cells locally at tumor sites. A phase 1 trial in patients with advanced ccRCC recently reported acceptable tolerability, with a response rate of 25% achieved in patients treated at target dose levels.^40^

## HIF2α development updates

Belzutifan, the first FDA-approved drug to inhibit the transcription factor hypoxia-inducible factor 2 alpha (HIF2α), was first approved in 2021 for RCC in the context of von Hippel-Lindau disease.^41^ It received another approval for advanced RCC in December 2023 based on the results of the LITESPARK-005 study, wherein belzutifan showed a consistent benefit across subgroups vs everolimus.^42^ Targeting a master regulator of angiogenesis, immune escape, and tumor metabolism, clinical development of belzutifan has continued in various combinations. Early success was seen in the phase 2 LITESPARK-003, which produced durable responses and some CRs when pairing belzutifan with cabozantinib in either first-line or subsequent line settings.^43^^,^^44^ Now, 2 phase 3 randomized trials have also reported positive results that may lead to new approvals. The double-blind LITESPARK-022 trial showed improved disease-free survival (DFS) for belzutifan + pembrolizumab vs placebo + pembrolizumab.^45^ LITESPARK-011, an open-label trial evaluating belzutifan + lenvatinib vs cabozantinib, also showed significant improvement in PFS for the combination.^46^

These positive results build further enthusiasm for the next wave of trials that will report in the future. Based on promising data from the KEYMAKER-U03 study, the phase 3 LITESPARK-012 trial evaluating a front-line triplet of pembrolizumab + lenvatinib + belzutifan vs pembrolizumab/quavonlimab (anti–CTLA-4) + lenvatinib vs pembrolizumab + lenvatinib has completed accrual (NCT04736706).^47^^,^^48^ The phase 3 LITESPARK-033 trial recently opened, and will test belzutifan + zanzalitinib vs cabozantinib in patients with disease recurrence following adjuvant immunotherapy (NCT07227402).

Other oral HIF2α inhibitors with a similar mode of action (allosteric inhibition of HIF2α’s PAS-B domain) have entered the clinic. The phase 1 ARC-20 study investigated casdatifan monotherapy or combination with cabozantinib in patients with advanced ccRCC. As monotherapy, casdatifan showed early signs of antitumor activity and responses in heavily pretreated patients across IMDC risk groups.^49^ With cabozantinib, activity was enhanced and tolerability remained manageable.^50^ Based on these results, the phase 3 PEAK-1 trial of casdatifan with cabozantinib is now enrolling (NCT07011719), and another pairing of casdatifan with the bispecific ICI volrustomig is on the horizon (NCT07000149).

Precision diagnostics could help guide treatment selection for HIF2α inhibitors. A radiolabeled molecule, [18F]PT2385 (a structural lead compound later developed into belzutifan), can be used for PET imaging of HIF2α levels in RCC tumor grafts.^51^ In an ongoing first-in-human clinical trial (NCT04989959), net influx of [18F]PT2385 was substantially higher in tumor than in healthy kidneys for 2 out of 3 patients. A second-generation tracer with improved target binding and pharmacokinetic properties, [^18^F]belzutifan, is now under development.

## Non-clear cell RCC subtypes

Molecularly defined RCCs are a new World Health Organization category as of 2022, with basis in findings from throughout the past decades. Kidney cancer displays tremendous morphologic and molecular heterogeneity at the tissue level, and variant RCC subtypes have divergent response to therapies.^52^ Molecular work-up may include tissue stains, immunohistochemistry, and various assays to detect genetic and/or molecular alterations, which are especially important when histology is ambiguous. Likewise, AI algorithms and liquid biopsy may soon enter the pathologist’s toolbox (*vide infra*).

Papillary RCC (pRCC) is the most common variant subtype of RCC, accounting for 10%-15% of kidney cancers. Approximately 80% of pRCC tumors are characterized by genome- or chromosome-level alterations resulting in upregulation of the MET signaling pathway.^53^ The phase 2 PAPMET trial showed cabozantinib to be a slightly favored option among TKIs, with a PFS benefit over sunitinib but no survival benefit.^54^^,^^55^ The phase 3 SAVOIR trial was underpowered to show benefit for the MET-specific inhibitor savolitinib as monotherapy vs sunitinib.^56^ Recently, more data from small trials has trickled in. Bevacizumab + erlotinib was an active combination in a phase 2 trial, particularly in patients with fumarate hydratase (FH)-deficient pRCC.^57^ Subgroup data from the phase 2 CALYPSO trial support durvalumab + savolitinib in MET-driven papillary RCC, which is being compared to both durvalumab and sunitinib monotherapies in the ongoing phase 3 SAMETA trial (NCT03091192).^58^ Another ongoing phase 3 trial in pRCC is STELLAR-304 (NCT05678673) comparing zanzalitinib + nivolumab to sunitinib. Both trials have finished accrual.

Chromophobe RCC (chRCC), the second-most common non-ccRCC variant, often presents as indolent with among the lowest mutational burden among human malingnancies.^59^ Lacking a clear oncogenic driver, chRCC features multiple whole chromosome losses and advanced tumors are depleted of T cells and unresponsive to most therapies, including ICIs.^60^ These tumors are also sensitive to oxidative stress and rely on ferroptosis resistance pathways, which increase metastatic potential via *TP53* mutations and sarcomatoid dedifferentiation.^61^ One target is FSP1 which suppresses ferroptosis.^62^^,^^63^ FSP1 is upregulated in chRCC and can be inhibited pharmacologically and synergistically with other drugs that induce ferroptosis.^64^^,^^65^ Another possible target is the tyrosine kinase receptor KIT which is highly expressed in chRCC vs other tumors and normal kidney, and can be targeted with an ADC.^66^ Clinically, combination therapies are producing better outcomes than monotherapies and it is therefore important to pursue combinations targeting multiple pathways for patients with chRCC.^67^

Translocation RCC (tRCC) is a rare subtype of kidney cancer, but has a much earlier age of onset than other variants and accounts for the majority of pediatric RCC diagnoses.^68^ Clinically it is an aggressive malignancy that often presents with metastases at diagnosis. Diagnosis itself is a challenge, necessitating more specialized fluorescence in situ hybridization or next-gen sequencing to detect TFE3/TFEB gene fusions.^69^ To rule out false negatives, TRIM63 assay can enhance diagnostic accuracy.^70^ TFE3 fusions drive changes in tumor metabolism toward oxidative phosphorylation, which potentially makes tRCC tumors sensitive to perturbations of this pathway.^71^ Identification of new targets in this malignancy is crucial, as one of the few trials in tRCC exclusively, a phase 2 study of axitinib + nivolumab vs nivolumab, was closed due to poor accrual.^72^

Sarcomatoid features, defined by the presence of any amount of atypical spindle cells resembling sarcoma, can co-occur in any molecularly-defined subtype of RCC and are present in roughly 5% of all cases. Biopsy is not accurate for identifying sarcomatoid features, which must be found in resected tumor after accurate gross examination. Sarcomatoid RCC has been historically associated with poor prognosis as patients present with advanced disease that responds poorly to chemotherapy and targeted therapy; however, outcomes are significantly improved on ICI-based therapy in both ccRCC^73^ and non-clear cell subtypes.^74^ Furthermore, a recent study subclassifying cases by proportion of sarcomatoid features found that patients whose tumors had ≥10% sarcomatoid histology had significantly worse survival and distinct transcriptomic perturbations vs those with <10%.^75^ At the transcriptional level, sarcomatoid RCC displays an inflamed phenotype with enrichment of pathways involved in immune response.^76^^,^^77^ Genomically, sarcomatoid components of ccRCC are enriched for mutations and deletions in *TP53*, *CDKN2A*, and the Hippo-YAP/TAZ pathway, some of which may be targetable with inhibitors currently in development.^78^ Finally, at the epigenomic level, FOSL1 was identified as a transcription factor that drives sarcomatoid dedifferentiation and response to ICI-based therapy.^79^

## Biomarkers and AI

AI tools for guiding treatment decisions are on the horizon. Using standard patient radiology imaging or pathology as inputs, machine learning and neural networks can help identify cryptic prognostic and predictive information that are present in the data. Improvements in detection through imaging have heightened detection of benign renal masses that would not warrant surgical intervention, creating an outstanding clinical need to differentiate benign renal masses from malignant and potentially aggressive RCC tumors in the perioperative setting. The benchmark for the preoperative prediction of tumor histology is the widely used R.E.N.A.L. nephrometry score-based nomogram.^80^ A deep neural network drawing on *n* = 300 preoperative CT scans demonstrated superior ability for predicting malignancy vs 3 trained human medical personnel.^81^ More recently, deep learning models were shown to outperform radiologists in an even larger sample (*n* = 4557) at both predicting malignancy and differentiating aggressive from indolent tumors.^82^ Notably, the AI algorithm automatically segments masses from the kidney structure, increasing efficiency by obviating the need for manual drawing of tumor boundaries. These models, developed on retrospective data, await prospective testing in a clinical trial setting.

Excision of the primary tumor generates another decision point where AI can help provide clarity. Clear cell RCC tumors are often histologically heterogeneous due to the asynchronous emergence of genetically distinct subclones. There are 9 architectural phenotypes of primary RCC tumor tissue that have divergent outcomes with respect to DFS,^83^ and classification into histopathologic subtypes also correlates with other known prognostic factors and clinical outcomes.^84^ This classification can be performed by pathologists with simple hematoxylin & eosin staining, from which deep learning algorithms can infer more convoluted features such as driver gene mutational status.^85^^,^^86^ This strategy was used to predict response to anti-angiogenic therapy in real-world and clinical trial cohorts about as well as a more elaborate RNA-based quantification method.^87^ Another multi-classifier deep learning model was able to predict recurrence of localized papillary RCC after surgery.^88^

Finally, machine learning has also been applied to sequencing data from tumor tissue and tested prospectively. Early results from clusters 1/2 the OPTIC RCC phase 2 trial (NCT05361720) were recently reported. Of 21 patients sorted for nivolumab/cabozantinib therapy who had at least one post-baseline scan, all experienced some reduction in tumor burden and none had PD as best response.^89^ Importantly, time from consent to sequencing, to analysis, and finally to treatment selection coalesced at 20 days, demonstrating the feasibility of an RNA biomarker strategy.

Collectively, these studies demonstrate that AI/deep learning can harness untapped information from both standard imaging and primary tumor tissue. Going forward, multimodal approaches that combine radiology, pathology, and sequencing data are most likely to provide a clinically useful biomarker.

## Liquid biopsy

Another noninvasive screening method is liquid biopsy, and the search for soluble factors in blood and urine has borne promising results. At the forefront is Kidney Injury Molecule-1 (KIM-1), a transmembrane glycoprotein overexpressed in ccRCC and pRCC; its extracellular domain can be detected from as little as 25 μL of urine or plasma.^90^ Plasma KIM-1 is associated with malignant pathology, worse metastasis-free survival, and higher risk of death,^91^ but also improved clinical outcomes on ICI vs placebo in an adjuvant study.^92^ Therefore KIM-1 is of interest both as a prognostic and predictive biomarker. Although urine sampling is more convenient and less invasive, KIM-1 levels in urine may vary due to non-tumor factors such as renal function or early kidney injury.^93^

Beyond KIM-1, ctDNA from plasma continues to be investigated. As powerfully shown in the adjuvant setting for muscle-invasive bladder cancer,^94^ ctDNA assays can lead to improved outcomes on the basis of real-time interventions. ctDNA can also be leveraged for sequential epigenomic profiling, which can reveal dynamic changes in transcriptional activity over time, including important RCC target genes like HIF2α.^95^ In the Kidney Cancer Research Consortium ctDNA study of patients with metastatic ccRCC on ICIs (NCT04883827), one cohort underwent serial plasma epigenomic profiling that identified increased promoter activity at RCC-associated genes in 6/7 non-responders and decreased activity in 4/4 responders,^96^ providing a promising early signal that this strategy can monitor tumor burden and tumor response to ICI treatment. This strategy can also aid in definitive diagnosis of tRCC, as the epigenomic signature of tRCC ctDNA is highly distinguishable from either ccRCC or healthy controls.^97^ Interestingly, the sarcomatoid epigenomic signature was detectable from circulating chromatin in patient plasma, paving the way toward a blood-based diagnostic test.^79^

## Conclusion

The coming years will likely see the continued integration of HIF2α inhibitor monotherapy (and likely combinations) into standard practice, and consideration must be paid to how this nascent era will impact perioperative decision making, drug resistance, subsequent therapy, and design of clinical trials with new drugs. While these larger-scale changes develop, breakthroughs in defining the tumor biology of all histologies of RCC must be pursued to realize personalized therapy from the adjuvant to systemic therapy settings.

KCRS is a crucial interface for the entire RCC research community to ensure fruitful research collaborations are proceeding with benefit for patients as a guiding principle. Many projects which have achieved success clinically, such as PCVs, were supported through the years by Department of Defense funding. Therefore, it was a major disappointment when the Kidney Cancer program was gutted entirely from the FY25 budget appropriations process. Funding in FY26 has returned, albeit at a lower level ($15M vs $50M in FY23/24),^98^ and this disruption should serve as a motivation to all stakeholders to redouble our efforts to advance research in service of our current and future patients.
